# Hospital Meetings, &c.

**Published:** 1899-06-10

**Authors:** 


### The Institutional Workshop.

### HOSPITAL MEETINGS, &C.

### NATIONAL HOSPITAL FOR PARALYSED AND EPILEPTIC.

Effects of the Prince's Fund.—The Attitude of Some Hospital Managers.

The biennial festival of the National Hospital for the Paralysed and Epileptic took place at the Hotel Metropole on Wednesday evening, May 31st, under the presidency of the Right Hon. Sir Charles Hall, K.C.M.G., Q.C., M.P. Those present included the Hon. Louis Greville, the Hon. W. F. B. Massey-Mainwaring, M.P., the Hon. Alban Gibbs, M.P., Colonel Sir Frederick Cardew, K.C.M.G., Governor of Sierra Leone, and Lady Cardew, Sir Gerald FitzGerald, K.C.M.G., Sir Henry Burdett, K.C.B., and Lady Burdett, Sir William Gowers and Lady Gowers, Sir Felix Simon, Mr. Lewis D. Hall, Sir James Crichton-Browne and Lady Crichton-Browne, Mr. Alfred L. Wheeler, Colonel and Sheriff Probyn, L.C.C., Mr. and Mrs. Colin Campbell, Dr. and Mrs. Buzzard, Dr. and Mrs. Ferrier, and Mr. Burford Rawlings, secretary and general director.

After the usual loyal toasts,

The Chairman proposed the toast of the evening, "Success to the National Hospital for the Paralysed and Epileptic." This festival, he said, was held with a two-fold object, and they had two big subjects to tackle. They wanted first of all to get sufficient money to pay off the difference between their revenue and expenses of maintenance; and, in the second place, they wanted, if they could, to fill up and bring to a conclusion the Commemoration Fund which was started in the Diamond Jubilee year of Her Majesty. He thought they ought to work very hard, especially for the latter object, because by so doing they paid a tribute of respect to that august lady, and they remembered the kindness with which she had always given her patronage and allowed the principal wing of the hospital building to be named after her late lamented son, the Duke of Albany. In a matter of this kind it was always necessary to look at the subject from a practical point of view, and as he was going to ask them to put their hands in their pockets he did not feel justified in doing so without giving them some very good and solid reasons. He turned therefore to the report published in April last, and called attention to the immense amount of work done by the hospital. Last year there were no less than 1,030 in-patients; the average number of beds occupied had risen to 176; and lastly there were over 6,000 out-patients treated, many of whom, of course, visited the hospital numbers of times. If that were not a good record he did not know what was. (Applause.) It was pleasant after studying those facts to see what had been said with regard to the work of the hospital by many illustrious and distinguished persons. He hoped all present had had an opportunity of reading a very interesting little brochure which had been prepared by their energetic secretary, entitled "Echoes of Many Voices." Mr. Burford Rawlings had done there about the best piece of work of its kind he (the chairman) had ever seen. (Applause.) He had not drawn upon his imagination for his facts nor upon his memory for his figures, but had recorded in a plain and unvarnished manner what had been said on various occasions in connection with this great charity by the distinguished men whose names he cited. The writer gave a summary of five reasons why they should all assist the hospital, and the majority of these were special reasons as affecting this particular institution. The first, and to his mind the most important, reason of all was the wide scope of the hospital—it was a national hospital. Patients came not only from all parts of Great Britain, but from Australia, the Cape, and Queensland. There was in connection with their hospital none of the "red tape" which was to be found in some places, although he was bound to say that on the whole their great hospitals were managed in a way worthy of admiration. (Hear, hear.) The hospital was open to every case of nervous disease or of brain disorder, from whatever distance it might come. They took in patients who were unfitted for the general hospitals, and to whom rest and quietness were of the utmost importance. Theirs was a hospital where those in charge had the opportunity of studying the patients, who were not distracted by the presence of patients who might be better in a general hospital. The source of knowledge which was developed by study which took place in the hospital was almost incalculable. Until a few years ago there was no combined deliberate attempt on the part of a large body like this to deal with the mysterious diseases of the brain. Hard-working benevolent men no doubt studied and dealt with them, but this was the first instance in which a hospital on a large scale had attempted to deal with those most distressing maladies. The pamphlet to which he had referred had been praised by a past-master in such matters—he referred to his friend, Sir Henry Burdett, whom he was glad to see there that night. (Applause.) They all knew the indefatigable work Sir Henry had done in connection with hospitals, in connection with the Prince of Wales's Hospital Fund, and with other charities, and praise from him was praise indeed. Sir Henry Burdett had written a letter in which he pointed out not only the important merits of the brochure, but also what they ought always to keep in mind with regard to this hospital, namely, that it was a special institution dealing with diseases of the brain coming from the pressure of brain work, and this would appeal to any man who had passed his life in a busy profession. There was one thing certain, and that was that they ought not to hide their light under a bushel. If they were to ask 90 per cent. of their friends in the West End it would be found they did not know that in a cosy little nook in Queen's Square, Bloomsbury, there was to be found a handsome, stately building fitted up with all the modern resources of civilisation so far as medicine and surgery were concerned, and that they did not know there existed a place where people could be sent and where their friends could go as contributing patients, and where they would have comfort such as they could not possibly get at home. He was privileged on the previous afternoon to go and see the wards with the sunlight pouring into the windows and the trees bursting into life outside, and where one could not even hear the busy hum of voices and the din of the City. He saw cheerful, contented faces, people of whom it was almost incredible to believe that they were suffering from serious mental malady. He urged those present to assist in making the hospital known among the many who at present knew nothing whatever about it. There was only one other matter to which he wished to refer, and which was also dealt with in Sir Henry Burdett's letter, namely, how was that hospital managed? Sir Henry said that "In management there is no hospital in London that holds a higher place." (Loud applause.) If they subscribed out of their generosity they might know they were giving money which would be devoted to the relief of deserving cases, and that they would thus be the means of helping not only to soothe the dying pillow, but to relieve from suffering many of those who, without such help, might pass lives of absolute misery and pain. (Applause.)

The toast was very enthusiastically honoured.

Sir Henry Burdett, K.C.B., said that certain writers who were constantly dealing with hospital questions habitually represented him as opposed to special hospitals, and one reason why he gladly valued the opportunity of coming there that evening to support this hospital was because it was a special hospital. But it was more than a special hospital, it was a hospital in deed and in truth. In fact, he might say of it that he did not think any thinking man or woman, being a worker, who had to depend upon brain-power for a living could pass over this hospital if they were worthy of their manhood or womanhood. (Applause.) It seemed to him that they owed it to themselves in this great City of London, the very centre of the greatest empire in the world, that their contributions, however small, should at any rate be governed by intelligence, because after all their hospital was an institution for the sustenance of the intelligence by restoring intellectual power. Therefore he said there was no intelligent worker in London, in the country, or indeed in the empire, who thought about the hospital and understood what it had done and was doing, who could in justice to his own intelligence refrain from giving something towards its support. (Applause.) The toast he had to propose was "The Board of Management." Whereas this hospital was essentially a brain hospital, he had the honour of proposing the health of the brains without the assistance of which it would be impossible for the institution to ever have existed, or for it to continue its progress day by day. There were committees of management and committees of management, and a great deal depended upon what kind of committee of management they had. In London their great difficulty had been, and was, to find men—and he thought it would be a good thing if they could find women—who would give of their intelligence by personal service, and would take part in the management of our hospitals, and so maintain their up-keep and prosperity. In the provinces, even when compared with the local honours, not omitting the office of Mayor, he believed he was right in saying that where there was marked intelligence in a provincial town there it was recognised that the highest honour to which a citizen could attain was to be selected by the governors of the local hospital to be a member of its board of management. He looked forward to the time—thanks in no small degree to the zealous interest which had been taken in our hospitals by the Royal family—when, through the new organisation, the League of Mercy, which it would take five or six years for the people to understand and to thoroughly grasp, there would be equal competition in London for the honour—and it was an honour and privilege—of having the right to give of one's self—not of one's money, but of one's self—to help to provide adequately for the relief of the sick. In dealing with a committee of management they had also to deal with those who worked under it. Matters would not go on adequately and properly if it were not for the staff, and he said with great satisfaction that in all his thirty years' experience and all his inspections of the hospitals in every country of the world he had never met anybody—and he was speaking now with an absolute personal knowledge of the man and his work, capacity, and powers—he had never met any man in the same office in any medical institution who could exceed in excellence their secretary and general director, Mr. Burford Rawlings. (Loud applause.) He thought it might be taken as understood that in drinking to the health of the board of management they drank also to the health of their secretary and director, their lady superintendent, their nurses, and all their humbler officials, from that most important person, the hall porter, downwards and upwards. (Applause.) In this connection he was very much gratified when he came into the room to find himself seated by a member of their board and to be told by him with great satisfaction that he had had the pleasure of knowing that some visitors had inspected their hospital as representatives of the Prince of Wales's Fund. Why was he pleased and delighted at that? Because they had yet something to seek in the way of popularity. Some of those who tried in their humble way to help those who were administering the affairs of the London hospitals by bringing more money to their treasury were visiting the institutions in order to make themselves familiar with the actual work done and the quality of that work, that they might justly apportion the contributions they were to receive from the Prince of Wales's Fund. They might be astonished, but he was speaking within his own knowledge of the facts, when he said there were hospitals where the visitors were not only not welcomed but where they were almost dreaded, and that there were officers who received the gentlemen in a spirit and manner incredible to him, as it must be incredible to all who witnessed it, even to those who were responsible for these officials. Now he would like that night, because he valued the opinion of the conductors of this hospital on account of the excellence of its management, and because it was a special hospital, to know whether they held that the Prince of Wales's Fund had injured their resources financially, or whether they recognised that by extending the area from which gifts came to the hospital, their income had benefited indirectly, as well as directly by the donation which they had received from the central fund? For his own part, as far as figures went to prove anything, having analysed carefully the whole of the accounts and the sources of income of the metropolitan hospitals of every description both before the Prince of Wales's Fund was instituted and since, he could find nothing but evidence of the good effect of the Fund upon the financial resources of every medical institution in the metropolitan area which was worthy of the name of hospital. (Applause.) He hoped he had not unduly transgressed from his toast by asking those who, after all, did benefit by some hundreds of pounds by the efforts which were being made to help to increase their support, whether they thought they were blind and irresponsible, or unnecessary evils, or whether they recognised those who were thus trying to help them as sane men working zealously outside their area, not in competition but in co-operation, desirous of extending the area of givers and to be successful in their efforts in so doing. He asked all present to drink very heartily to the health of the board of management and the officers who worked under them. (Applause.)

The Hon. Louis Greville, in acknowledging the compliment in the unavoidable absence of the treasurer (the Hon. H. D. Ryder), assured those present that every endeavour would be made on the part of the board to justify the confidence that had been reposed in them.

Sir James Crichton-Browne, in submitting "The Medical and Surgical Staff," remarked that this national hospital possessed a galaxy of medical and surgical talent of which it might well be proud.

Dr. Buzzard having briefly responded,

Mr. Burford Rawlings, the Secretary, said that, in reply to Sir Henry Burdett's appeal as to what they considered the influence of the Prince of Wales's Fund to be, he answered unhesitatingly and without any reservation whatever, that so far as his feeling was concerned, he regarded the influence of the Fund as wholly beneficial. He said this not only having regard to the assistance rendered by the Fund, but also to the careful but by no means inquisitorial investigation of the affairs of the hospitals which preceded that assistance being granted. He had also in his mind that there were certain hospital managers—he hoped only a few—who were not altogether pleased with the Prince's Fund. He confessed he was not able to understand their attitude, because to those who were acquainted with these matters two facts stood out prominently. The first was that up to the present time only a very small fraction of the population of all classes had contributed anything in support of the hospitals; and secondly, that all the efforts of individual institutions to enlarge the area from which subscriptions were drawn had signally failed. The Prince of Wales's Fund was endeavouring to remove this, and to teach the lesson that the maintenance of the hospitals was not a luxury to be enjoyed only by the generous few, but a duty to be shared by all, and he could not help thinking that, if only to the extent that the managers of the Fund were able to enforce that lesson, they were entitled to the profound gratitude of every hospital manager. He had hoped to hear from Sir Henry Burdett something about the future of the Fund, to which subject he did not think Sir Henry alluded. He (the speaker) hoped there was a great future for it, and certainly in estimating the value of the Prince's Fund they must not consider merely the figures of the present but must also think of its infinite possibilities in the future. With regard to their own hospital he wished to say they were at present receiving from the Fund an annual subscription of £800, and in addition to that they had received a grant for special purposes of something like a similar amount. On the other hand, he did not know of any single subscription which had been withdrawn because of the existence of the Fund. (Loud applause.) As he understood it the Prince's Fund was intended not to supplant but to supplement and even to encourage the energies which were at present being devoted to the hospitals, and he thought no one would admit more readily than Sir Henry Burdett himself that the hospitals as they stood were largely indebted to the active exertions of individual workers, and that there would always remain for the exercise of those virtues a devotion and self-sacrifice without which he believed the voluntary system would long ago irremediably have perished. (Hear, hear.) As to the visit of the delegates of the Prince's Fund to the hospital the other day, he might say that that visit had none but pleasant memories for those connected with the institution. One of the delegates, as he was leaving, said "I suppose we are among the best hated people in London" (meaning his colleagues and himself). He had alluded to the fact that there was a difference of opinion among hospital managers, and perhaps that might be considered justifiable, or at any rate reasonable, but what he could not understand was that any institution should accept the substantial assistance afforded by the Prince's Fund and be at no pains to conceal its want of appreciation and gratitude. (Hear, hear.) He would not have ventured upon that remark, although it was entirely his personal view, if he did not think that the one thing which was wanting to make the Prince's Fund the success it ought to be was the cordial support and loyal co-operation of the hospitals themselves. (Hear, hear.) The Secretary then announced the list of donations, the chairman's list amounting to £2,637 3s. 6d., and the grand total being £2,807 17s. 6d.

The toast of "The Chairman" was proposed by the Hon. Alban Gibbs, M.P., and Sir Charles Hall, in reply, said he had heard with great pleasure what Sir Henry Burdett had said with regard to the Prince's Fund, and also the remarks made by the general director in answer to those observations. There was one fact that stood out pre-eminent, and which no one who had studied the question could fail to have observed, and that was that the Fund had tapped sources which were never tapped before. (Hear, hear.) It was not a question of taking money away from the subscribers to hospitals, but it was well known to anyone who had made the slightest inquiry into the matter that the Fund had brought before thousands of people in the country who had never subscribed before the merits of the hospitals and their duty to subscribe to them. (Applause.)

The proceedings were considerably enlivened by an excellent musical programme, there being vocal selections by Madame Belle Cole, and instrumental (piano) by Miss Janotha (hon. life governor of the hospital), and the "Bijou" Orchestra, conducted by Mr. J. Pougher.

### PADDINGTON GREEN CHILDREN'S HOSPITAL.

The annual meeting of the Paddington Green Children's Hospital was held on Thursday, June 1st, at the institution, Colonel the Hon. Francis C. Bridgeman presiding. In their report for 1898, which was presented, the committee stated that the buildings having been completed so far as was at present practicable, they had devoted their energies to strengthening the financial position and to improving the general work of the hospital. With the help of a large legacy efforts in the first direction had been eminently successful. Not only could the committee show that all the moneys received for the perpetual endowment of cots were duly invested, but also that a substantial reduction had been effected in the debt due to their bankers, to whom, however, £1,300 was still owing. In the second direction various new appliances for the use of the medical and surgical staff had been provided; but there was badly wanted a passenger lift to convey patients from floor to floor. This, the committee thought, should not be a charge on the general funds of the hospital. The medical and surgical staff had strongly recommended the provision of the lift, for which the necessary space existed in the building. It therefore only remained to purchase and fix the lift itself. The cost would not exceed £400, towards which £120 had been promised. The committee noted with pleasure the continuance of the increase in their reliable sources of income. Legacies had realised £4,400; and special thanks were accorded in the report to Messrs. Barnato Brothers, the executors of Mr. Woolf Joel, who apportioned £250 to the hospital under Mr. Joel's will and paid the legacy duty themselves. The Prince of Wales's Fund had forwarded a special donation of £150 to the hospital, and one of £25 to the convalescent home, whilst £100 had been received from the trustees of Smith's (Kensington Estate) Charity and £24 came as a donation from a former Paddington resident in Singapore, the amount being the proceeds of some performances given there by children. The committee further acknowledged the generous support again given them by the congregation of Christ Church, Lancaster Gate, who sent £243, the proceeds of offertories, and the report referred warmly to the support given the hospital by the congregations of St. Matthew's, Bayswater, and St. Stephen's Church, Westbourne Park. All the annual subscriptions for cots had been renewed, while an additional subscription to maintain a cot had been received. Miss Edith Rendel had continued her valuable help to the institution by receiving a few of the children in her Convalescent Home at Charlwood. The Convalescent Home at Whealdstone, near Harrow, was under the charge of Nurse H. Norton, and the committee reported that it continued to be a valuable adjunct to the hospital, 77 children having been sent there during the year. The pecuniary position, however, remained a source of much anxiety to the committee, as it received very inadequate support, the actual subscriptions being only £41, whilst the expenses were £515. The cases admitted to the hospital during the year were 563, which, with 38 cases remaining from 1897, gave a total of 601; of these, 485 were discharged cured and relieved, 78 died, and 38 cases remained. New out-patients numbered 11,647. The total ordinary income amounted to £3,087, and the total ordinary expenditure was £3,443, whilst the total extraordinary income was £6,082, and the total extraordinary expenditure, £888, there remaining cash balances of £1,166; amounts invested, £2,959; and repayment to bankers of £1,600. The Chairman, in moving the adoption of the report and accounts, said they must all feel that the report on the whole was a most satisfactory one. With regard to the provision of a lift, they had since received sufficient to cover the cost of its erection and purchase except about £21; it had been ordered, and the work would be put in hand at once. The least satisfactory part of the report was that relating to the convalescent home, to which the subscriptions had been very small, whilst it was not pleasing to have money owing to the bankers. He trusted they would receive increased support. The motion was seconded by Mr. Henry Seton-Karr, M.P., and unanimously adopted. Lieut.-General Lowry moved the re-election of a number of members of the committee, which was seconded by Mr. T. A. Denny, and agreed to, after which votes of thanks were heartily accorded the committee, the hon. secretary, and the medical staff, and the proceedings terminated.

### HOSPITAL FOR SICK CHILDREN.

The annual court of governors of the Hospital for Sick Children, Great Ormond Street, was held in the board-room of the hospital on Thursday, May 31st, Lord Kinnaird presiding. The forty seventh annual report stated that the number of in-patients, which amounted to 2,067, showed an increase of 121 over last year's total. Out-patients numbered 24,249, an increase of 576 since 1897, while 89,220 was the total number of attendances; 238 patients were sent to Cromwell House, the convalescent home at Highgate. As the receipts were only £9,004, while the expenditure amounted to £15,131, the accounts for the year showed a deficit of £6,127. To meet this part of the £9,514 received from legacies was applied. The committee greatly deplored the necessity for this use of a most precarious source of income. Subscriptions amounted to £2,718, and donations, which in 1897 totalled £2,079, showed a decrease of £838. The committee announced with gratitude the receipt of £500 from the Prince of Wales's Fund. This sum was given to facilitate the opening of a whooping-cough ward. One-third of the purchase-money for the recently-acquired hospital of St. John and St. Elizabeth had been delivered, and there now remained to be paid the sum of £20,000. The interior of the building, which was formerly a convent, had been entirely rearranged, and as the provision of a separate room for each nurse was now possible, it could not fail to add largely to the comfort and health of the nursing staff. Inquiries made in several cases into the circumstances of out-patients resulted satisfactorily, and showed that the hospital suffered little from abuse in this direction. The endowment of six cots in the course of the year was announced with great pleasure by the committee. Mr. Edmund Owen, the senior surgeon, had retired from the active staff, and the committee desired to record their appreciation of his valuable services. To fill the vacancy created by the resignation of Mr. Frank C. Madden, F.R.C.S., of the resident staff, Mr. B. Dyball, F.R.C.S., was elected. Lord Aberdare is announced as a new member of the committee. The friends and supporters of the hospital are urgently appealed to for help in paying off the residue of the purchase money for the nurses' home. In conclusion, the committee state that unless the public are less apathetic with regard to the welfare of the hospital, its work cannot be carried on. Mr. Arthur Lucas (chairman of the committee of management) referred in his speech to the disheartening financial result of the year's work as a disgraceful state of things. For several years past the work of the hospital had gone on steadily increasing, while the annual revenue as steadily diminished. The apathy of the public he could only believe was due to ignorance of the facts. If matters did not improve he saw but two alternatives—to close some of the wards or to throw the burden of the support of the hospital upon the rates. This latter proceeding would inevitably dam the flow of public benevolence, and cause the management of the hospital to fall into the hands of a lower class of men. Mr. Lucas remarked that it was not only the poor who had reason for gratitude towards the hospital. In providing a most valuable training for the medical profession it conferred indirect benefit upon all classes. After the adoption of the report, the officers and the members of the committee for the new year were elected. Votes of thanks having been passed to the medical staff, the honorary auditors, and to Lord Kinnaird for presiding, the meeting closed.

